# Synchronous primary pulmonary adenocarcinoma and extranodal marginal zone lymphoma of mucosa-associated lymphoid tissue: A case report and literature review

**DOI:** 10.1097/MD.0000000000020865

**Published:** 2020-07-17

**Authors:** Lixia Sun, Bing Zhang, Ke Xuan, Li Qi, Jingjing Wang, Quan Li, Jianwei Liu, Yubo Wang, Liping Sun, Xiaomei Li, Hong Ji

**Affiliations:** a Department of Pathology; b Department of Surgery, Binzhou Medical University Hospital, Binzhou; c Department of Pathology, Huantai Renmin Hospital, Huantai; d Department of Gastroenterology, Binzhou People Hospital; e Department of Radiology, Binzhou Medical University Hospital; f Department of General Practice, Haifeng Street Health Service Center; g Center for Disease Control and Prevention of Shandong Binzhou, Binzhou, China.

**Keywords:** extranodal marginal zone lymphoma of mucosa-associated lymphoid tissue, pulmonary adenocarcinoma, pulmonary lymphoma, synchronous tumor

## Abstract

**Rationale:**

Synchronous pulmonary lymphoma and carcinoma is relatively rare. And synchronous pulmonary lymphoma and adenocarcinoma in the same site is extremely rare.

**Patient concerns:**

We presented a 69-year-old female with a tumor mass in right upper lung.

**Diagnosis:**

Pathological and immunohistochemical findings revealed lung adenocarcinoma and extranodal marginal zone lymphoma of mucosa-associated lymphoid tissue.

**Interventions:**

The patient received thoracoscopic guided right upper lobectomy and focal lymph node dissection after systemic anesthesia. Besides, 6 cycles of chemotherapy were given based on meprednisone, gemcitabine and cisplatin in local hospital.

**Outcomes:**

In the 12-month follow-up, the patient was still alive with no local recurrence, metastasis and lymph node involvement.

**Lesson:**

A comprehensive literature research was performed, and 6 cases of synchronous pulmonary lymphoma and adenocarcinoma in the same site and 10 cases in different sites were identified since 2000. Most patients with synchronous pulmonary lymphoma and carcinoma were middle-aged and elderly with the median age was 64 years presenting a male predisposition. The most frequent type of primary pulmonary lymphoma was B-cell non Hodgkin lymphoma, especially mucosa-associated lymphoid tissue lymphoma, and the lung cancer is predominantly adenocarcinoma.

## 1 Introduction

Lymphoma occurs in many organs of human beings. To date, rare studies have been focused on synchronous lymphoma combined with other tumors.^[1–3]^ In particular, the occurrence of lymphoma in the same nodule of a certain organ is exceedingly rare. According to the previous literatures, there are only 6 cases presenting with synchronous lymphoma and adenocarcinoma in the same pulmonary site^[1–6]^ and 10 cases in different sites.^[7–16]^ In this study, we reported a 69-year female patient showing a rare pulmonary collision tumor consisted of extranodal marginal zone lymphoma of mucosa-associated lymphoid tissue (MALT lymphoma) and adenocarcinoma in a single site.

## 2 Case report

**Figure 1:**
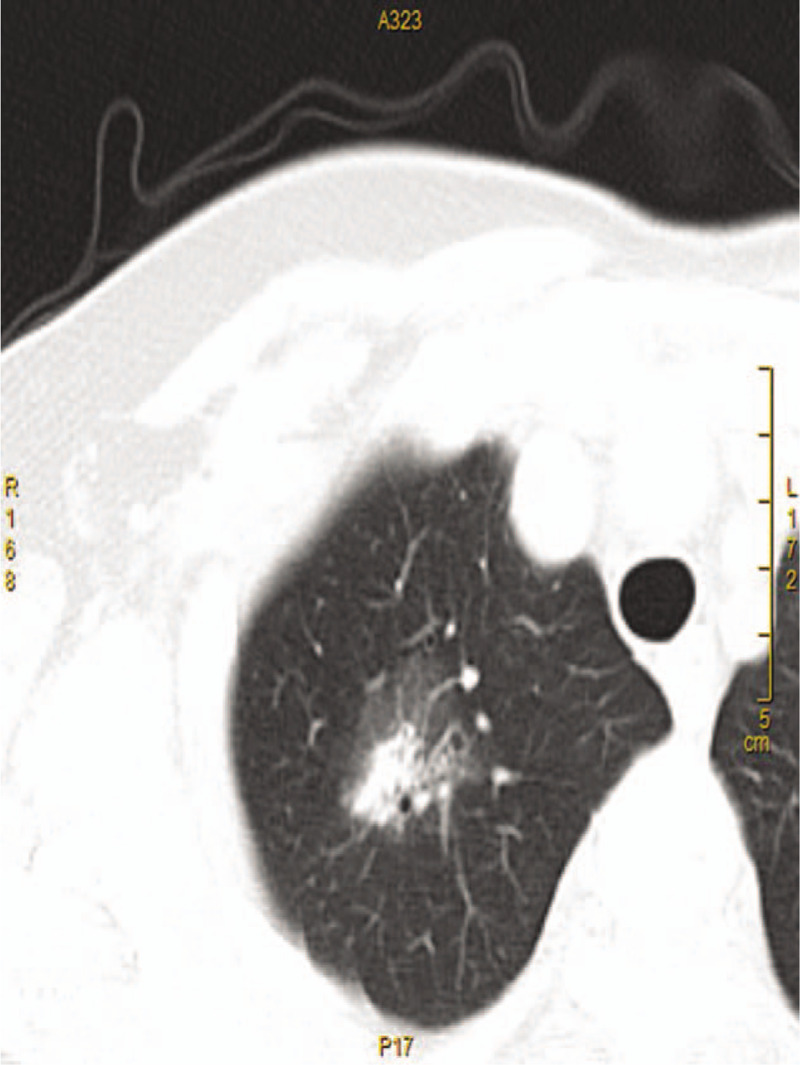
Thoracic computed tomography scan demonstrated a mass (2 cm) in upper lobe of right lung.

A 69-year-old female presented to our hospital with a complaint of headache, dizziness, cough, night sweats, and weight loss on May 30, 2018. The physical examination findings were normal. All routine laboratory test results including routine hematological examination and serum chemistry were in the normal ranges. Computed tomography (CT) revealed a soft tissue lesion in the chest with a maximal diameter of 2 cm, which was localized in the upper lobe of the right lung with a spiculated contour (Fig. 1). There was no hilar or mediastinal lymphadenopathy. The abdominal and pelvic CT scan findings were normal. She received cholecystectomy in 2000 and partial thyroidectomy in 2008, respectively. In addition, she reported a past medical history of hypertension. A smoking history was denied. Thoracoscopic guided right upper lobectomy was performed after systemic anesthesia, and focal lymph node dissection (group 4, 7, and 10) was given on June 2, 2018.

Macroscopically, the specimen obtained from the lobectomy was in a size of 11.5 cm× 8.5 cm× 2.5 cm, which included a lesion (2 cm×1.8 cm× 1.5 cm) in apical segment of right superior lobe. There was no pleural invasion. The tumor border was not clear. The cut surface was in white-and-black color, of a slightly hard texture without necrosis.

**Figure 2:**
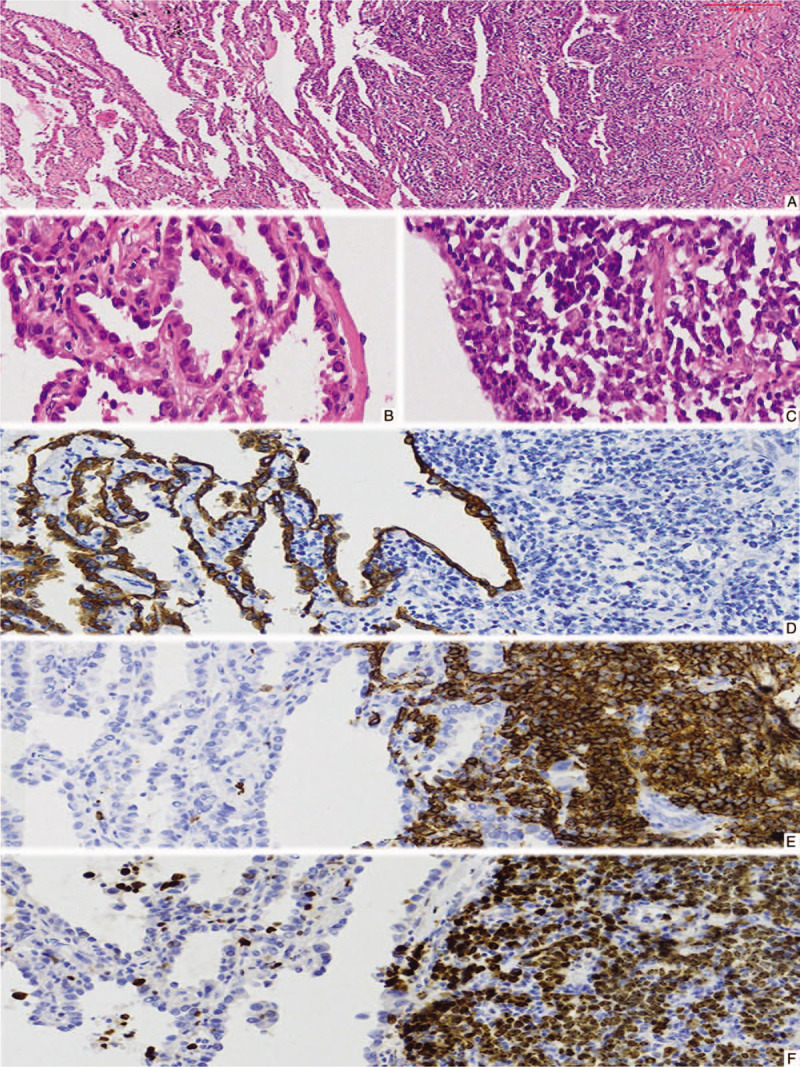
The tumor component containing adenocarcinoma (left) and mucosa-associated lymphoid tissue (MALT) lymphoma (right) (A, HE staining, 60×); adenocarcinoma with a mixed lipidic and papillary pattern (B, HE staining, 400×) around the MALT lymphoma (C, HE staining, 400×); adenocarcinoma was positive for CK (D, left, Enlivision, 200×), negative for CD20 (E, left, Enlivision, 200×) and low ki-67 index (F, left, Enlivision, 200×), while MALT lymphoma was negative for CK(D, right, Enlivision, 200×), positive for CD20 (E, right, Enlivision, 200×) and high ki-67 index (F, right, Enlivision, 200×).

Microscopically, there were 2 different areas (Fig. 2A). An invasive adenocarcinoma was identified in the peripheral part of the mass. It was a lepidic adenocarcinoma (Fig. 2B), which mixed with papillary and acinar pattern. The lymphoid cells were diffusely infiltrated into the central part of the lesion and admixed with the adenocarcinoma, which were small to medium-sized, presenting with slightly irregular nuclei (Fig. 2C). The texture of chromatin was moderately coarse with inconspicuous nucleoli. Additionally, the other lymphocytes, plasma cells, and histocytes were also shown. Some larger cells were scattered. Lymphoepithelial lesions were found in the bronchial mucosa. In the borderline area, there were acinar and papillar adenocarcinoma in the lymphoid cells, and the lymphoid cells extended along the stroma lined by adenocarcinoma cells. Immunohistochemically, the tumor cells of the adenocarcinoma component were positive for CK (Fig. 2D) and negative for CD20 (Fig. 2E), CD3, CD5, CD43, BCL2, CD30, CD23, CD10, and cyclin D1. The Ki-67 index was low (Figure 2F). The lymphoid cells were positive for CD20 (Figure 2E) and negative for CK (Figure 2D), CD3, CD5, CD43, BCL2, CD30, CD23, CD10, and cyclin D1. The Ki-67 index was higher (Fig. 2F).

The patient was finally diagnosed with lepidic-predominant lung adenocarcinoma (stage T1bN0M0, WHO 2014) associated with a low grade B-cell lymphoma of mucosa-associated lymphoid tissue type (stage I-E, Ann Arbor). There was no pleural involvement or lymphatic metastasis. For the treatment, the patient received 6 cycles of chemotherapy using meprednisone (80 mg, d1-5), gemcitabine (1.6 mg, d1–d8) and cisplatin (30 mg, d1; 40 mg, d2-3) in local hospital. She was still alive in the 12-month follow-up with no local recurrence, metastasis and lymph node involvement. The treatment outcome was classified as complete response according to the response criteria of the International Working Group (IWG) (Cheson classification).

## 3 Discussion

Primary pulmonary lymphoma (PPL) is defined as mono-clonal lymphocytic infiltration of the lung with or without hilar lymph nodes involvement upon diagnosis or up to 3 months thereafter. PPL is a very rare disease with an incidence of 0.5%–1.0% among lung malignancies and 0.4% among lymphomas.^[17]^ The most frequent type of PPL is B-cell non Hodgkin lymphoma (B-NHL), especially the low-grade mucosa-associated lymphoid tissue type with an incidence of 70% to 90%. The second most common type is primary pulmonary diffuse large B-cell lymphoma (DLBCL), which accounts for 10% of primary pulmonary NHL.^[18]^ In clinical settings, these patients usually present with non-specific symptoms. In the radiological aspect, it is extremely difficult to distinguish such disease from more common lung malignancies (e.g., bronchogenic carcinoma).

Rare patients show coexistence of lymphoma and other tumors in the same or different anatomical sites. According to the previous studies, cases of synchronous lymphoma and other neoplasms in the same organ showed involvement in stomach, thyroid, kidney, throat, and tonsils.^[10,19–21]^ Rare studies have been published on cases showing lymphoma and carcinoma in the lung tissues.

Table 1 summarized the published cases of synchronous pulmonary lymphoma and carcinoma. There were 17 cases (male: 10; female: 7) including our case. Most patients were middle-aged and elderly with the median age was 64 years. All the 17 cases had no specific symptoms. Eight patients (male: 5; female: 3) had a history of smoking. Among these patients, 15 were adenocarcinoma and 2 were diagnosed with squamous cell carcinoma. In the 15 cases of synchronous adenocarcinoma and lymphoma, the histological types of lung cancer included lepidic predominance (n = 2), acinar (n = 1), papillary (n = 2), mixed of adenocarcinoma (n = 2), middle-low differentiated adenocarcinoma (n = 1) and unclassified adenocarcinoma (n = 7). Among the 17 cases, 15 were diagnosed with B-NHL, 1 with T-cell lymphoma and 1 with unclassified lymphoma. The most frequent type was MALT lymphoma (6/17, 35.3%), followed by mantle cell lymphoma (MCL)(4/17, 23.5%), DLBCL (4/17, 23.5%) and diffuse B-cell lymphoma (1/17, 5.9%). There was only 1 cases of T cell lymphoma, which was lymphoepithelioid cell lymphoma, also known as Lennert lymphoma. From the summary, there were only 7 cases (male: 4; female: 3; mean age: 61 years;) with synchronous carcinoma and lymphoma in the same nodule of the lung, together with our cases. They were all confirmed with adenocarcinoma. Two males and one female showed a smoking history. The histological types included lepidic predominance (n = 2), mixed adenocarcinoma (n = 1) and unclassified adenocarcinoma (n = 4). Four were diagnosed with MALT lymphoma (57.1%), 1 with mantle cell lymphoma (14.3%), 1 with DLBCL (14.3%), and 1 with lymphoepithelioid cell lymphoma (14.3%). Taken together, most patients with synchronous pulmonary lymphoma and carcinoma are middle-aged and elderly, and it is more common in men than women. The most frequent type of PPL is B-NHL, especially MALT lymphoma, and the lung cancer is predominantly adenocarcinoma.

**Table 1:**
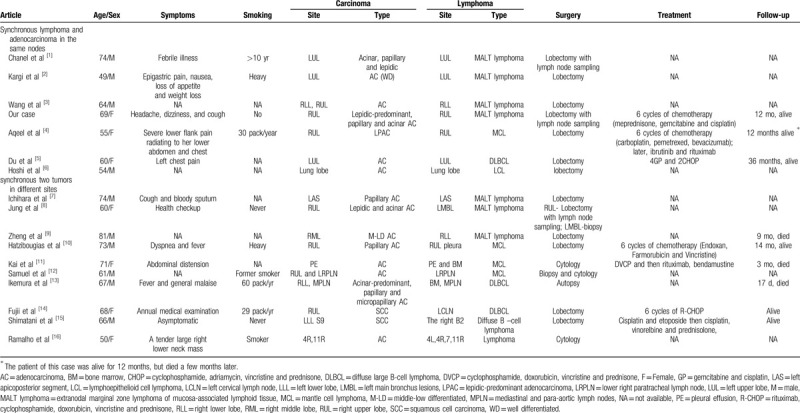
Key characteristics of patients with synchronous lung carcinoma and lymphoma.

Collision tumors are rare entities defined by the presence of 2 tumors of independent origins within the same specimen.^[22]^ It is distinguishable from tumors containing 2 or more cell lines arising from a common source. In addition to the same site, collision tumors can also occur within adjacent organs or in conjunction with a systemic malignancy or as a metastatic phenomenon. To date, 5 aspects have been reported to be associated with the pathogenesis of collision tumors. First, alteration of regional microenvironment induced by an already present tumor may lead to increased risk of secondary tumor.^[23]^ For example, accumulation of inflammatory cells caused by existence of 1 type of cancer can promote cell proliferation, which is an important pathogenesis of malignancies. Secondly, 2 malignant cells in collision tumor may originate from carcinogenesis of a common stem cell. In this case, the possibility of lung adenocarcinoma and DLBCL from the same stem cells was low as the 2 types of tumor cells were originated totally from different progenitor cells. Thirdly, it has been well known that gene mutation (e.g., RAS mutation) may be associated with the pathogenesis of several tumors. Overexpression of IL-17A and CD70 gene in a collision tumor consisted of primary laryngeal mucosal melanoma and invasive squamous cell carcinoma was reported.^[24]^ Fourthly, carcinogenic risk factors (e.g., smoking and aging) may ultimately trigger in the pathogenesis of multiple primary cancers synchronously.^[25]^ Finally, the synchronous onset of 2 different malignancies in the same site was a random coincidence, which showed no specific relations among these lesions.^[13]^

The incidence of synchronous pulmonary lymphoma and carcinoma is extremely low. Although diagnosis can be given by broncho-scopic or transbronchial biopsy, or percutaneous needle biopsy, there might be possibilities of misdiagnosis. Therefore, in the presence of many lymphoid cells and adenocarcinoma cells in the same nodule, much attention should be paid to the pathology findings to present misdiagnosis. For these tumors, preoperative diagnosis is difficult, and complete tumor resection is required for the diagnosis of the different components. There is yet no standard or guideline for the tumor grade, stage, treatment, and prognosis information. The treatment of these synchronous neoplasms is complex, including observation, surgery, radiotherapy or chemotherapy alone or in combination. Surgical resection and radiotherapy are preferred for cases with localized lesions, and chemotherapy may be considered for bulky or disseminated cases. In our case, the patient underwent a combination of treatment including surgical excision and chemotherapy, who presented satisfactory healthy conditions in the 12-month follow-up. In Table 1, there were 13 cases with lobectomy including our case, and 6 of them received chemotherapy. All were alive in the follow-up. To be specific, Du et al^[5]^ reported that lung adenocarcinoma and lymphoma were well controlled in certain cases after 4 cycles of chemotherapy with gemcitabine, cisplatin (GP) regimen, as well as 2 cycles of chemotherapy using cyclophosphamide, adriamycin, vincristine, prednisone (CHOP) regimen. No recurrence was observed within 3 years. Shimatant et al^[15]^ reported systemic chemotherapy using cisplatin and etoposide was effective for carcinoma and lymphoma, which was proved to show complete remission of lymphoma by bronchoscopy cytologically and pathologically. Fujii et al^[14]^ performed therapy for malignant lymphoma earlier than lung cancer. However, there are few treatment options for these patients. It is still not certain whether the treatment of lung lymphoma is prior to lung adenocarcinoma or not. In this study, the patient received 6 cycles of chemotherapy using meprednisone, gemcitabine, and cisplatin. She showed no recurrence and new lesion in the 12-month follow-up. Due to the limited published data, more cases are required to summarize the key diagnosis and therapeutic selection of the synchronous neoplasm.

## Author contributions

**Investigation:** Lixia Sun, Ke Xuan, Qi Li, Jingjing Wang, Quan Li, Jianwei Liu, Yubo Wang, Liping Sun, Xiaomei Li.

**Writing – original draft:** Lixia Sun, Bing Zhang.

**Writing – review & editing:** Hong Ji.
